# Heavy Metal Soil Contamination Detection Using Combined Geochemistry and Field Spectroradiometry in the United Kingdom

**DOI:** 10.3390/s19040762

**Published:** 2019-02-13

**Authors:** Salim Lamine, George P. Petropoulos, Paul A. Brewer, Nour-El-Islam Bachari, Prashant K. Srivastava, Kiril Manevski, Chariton Kalaitzidis, Mark G. Macklin

**Affiliations:** 1 Faculty of Natural Sciences, Life and Earth Sciences, University Akli Mohand Oulhadj of Bouira, 10000 Bouira, Algeria; 2 Department of Geography and Earth Sciences, University of Aberystwyth, Ceredigion, Wales SY23 3DB, UK; pqb@aber.ac.uk; 3 Department of Soil and Water Resources, Institute of Industrial and Forage Crops, Hellenic Agricultural Organization “Demeter” (former NAGREF), Directorate General of Agricultural Research, 1, Theofrastou St., 41335 Larisa, Greece; petropoulos.george@gmail.com; 4 School of Mineral and Resources Engineering, Technical University of Crete, Kounoupidiana Campus, 73100 Crete, Greece; 5 Faculty of Biological Sciences, University of Sciences and Technology Houari Boumediene, BP 32, El Alia, Bab Ezzouar 16111, Algeria; bacharinouri@gmail.com; 6 Institute of Environment and Sustainable Development & DST Mahamana Center for Excellence in Climate Change Research, Banaras Hindu University, Varanasi 221005, India; prashant.just@gmail.com; 7 Department of Agroecology, Aarhus University, Blichers Alle 20, 8830 Tjele, Denmark; kiril.manevski@agrsci.dk; 8 Department of Geoinformation in Environmental Management, Mediterranean Agronomic Institute of Chania, 73100 Crete, Greece; chariton@maich.gr; 9 School of Geography, College of Science, University of Lincoln, Brayford Pool, Lincoln, Lincolnshire LN6 7TS, UK; mmacklin@lincoln.ac.uk

**Keywords:** hyperspectral data, heavy metals, floodplain, soil spectral library, regression modelling

## Abstract

Technological advances in hyperspectral remote sensing have been widely applied in heavy metal soil contamination studies, as they are able to provide assessments in a rapid and cost-effective way. The present work investigates the potential role of combining field and laboratory spectroradiometry with geochemical data of lead (Pb), zinc (Zn), copper (Cu) and cadmium (Cd) in quantifying and modelling heavy metal soil contamination (HMSC) for a floodplain site located in Wales, United Kingdom. The study objectives were to: (i) collect field- and lab-based spectra from contaminated soils by using ASD FieldSpec^®^ 3, where the spectrum varies between 350 and 2500 nm; (ii) build field- and lab-based spectral libraries; (iii) conduct geochemical analyses of Pb, Zn, Cu and Cd using atomic absorption spectrometer; (iv) identify the specific spectral regions associated to the modelling of HMSC; and (v) develop and validate heavy metal prediction models (HMPM) for the aforementioned contaminants, by considering their spectral features and concentrations in the soil. Herein, the field- and lab-based spectral features derived from 85 soil samples were used successfully to develop two spectral libraries, which along with the concentrations of Pb, Zn, Cu and Cd were combined to build eight HMPMs using stepwise multiple linear regression. The results showed, for the first time, the feasibility to predict HMSC in a highly contaminated floodplain site by combining soil geochemistry analyses and field spectroradiometry. The generated models help for mapping heavy metal concentrations over a huge area by using space-borne hyperspectral sensors. The results further demonstrated the feasibility of combining geochemistry analyses with filed spectroradiometric data to generate models that can predict heavy metal concentrations.

## 1. Introduction

The United Kingdom (UK) Environment Agency has listed over 1300 former mining sites responsible for heavy metal contamination of both land and water [1,2]. River systems can become contaminated by metals, for example lead (Pb), zinc (Zn), cadmium (Cd) and copper (Cu) if their drain catchments are underlain by mineralised geologies. In the UK, peak base-metal mining activity occurred in the 18th and the 19th centuries, when there was little or no environmental legislation preventing the release of contaminated water and sediments into the water courses. Floods are involved directly as serious agents of contaminant dispersion [3,4,5], resulting in sedimentation on agricultural and residential lands, where contaminants may remain for 10s or 100s of years until they are remobilised via surface or river bank erosion. Contaminated floodplain soils and sediments pose a potential danger to human health, safety of agricultural products and may adversely affect the environment [2,6]. 

Considering the paramount importance of soil for food security and the increasing size of urbanisation, it is important to identify and manage metal contaminated sites [7,8,9]. Therefore, an understanding of the contamination risk is required, as well as the development of quick, feasible and affordable estimation methods [10,11,12,13]. Traditional techniques for evaluating metals contamination in the environment typically involve field-based soil/sediment sampling, wet chemical digestion and subsequent laboratory analysis, followed by interpolating outputs to create spatial risk maps [14,15,16]. However, such approaches are time-consuming and often very expensive [2,17,18,19]. Advances in hyperspectral remote sensing are increasingly being applied in metal soil contamination studies, providing a more rapid, cost-effective and spatially extensive way to map contamination [20,21,22,23,24].

The utility of hyperspectral imaging to map the distribution of heavy metals in mining regions has previously been demonstrated by several studies [25,26,27,28,29,30]. Soil properties and concentration of minerals can be determined using hyperspectral imaging techniques since these are able to provide spectrally-rich and spatially-continuous information that can be extended for mapping and monitoring of soil contamination. Reflectance spectroradiometry is relatively more cost-effective than traditional measurements based on chemistry [17,31,32,33,34]. 

Spectral signatures obtained from soil constituents are distinguished based on their reflectance in specific bands of the electromagnetic spectrum [35,36,37]. Visible (VIS, 350–800 nm), near infrared (NIR, 800–1350 nm) and shortwave infrared (SWIR, 1350–2500 nm) spectroradiometers are used largely in soil science, since they can be handled easily in the field. After correct calibration, they can be used to estimate several soil properties such as total carbon and nitrogen, sand and clay contents, cation exchange capacity and pH (e.g., [38,39]). Schwartz et al. [40] summarises the application of VNIR reflectance for estimating the soil contamination, and Shi et al. [41] review the role of VNIR soil spectra for predicting concentration of heavy metals.

This study aims at investigating the potential added value of field spectroradiometry when combined with geochemical analyses of Pb, Zn, Cu and Cd, to quantify and model heavy metal soil contamination (HMSC). The specific objectives are to: (i) collect field- and lab-based spectra from contaminated soils and build associated spectral libraries; (ii) identify the specific spectral intervals associated with the modelling of HMSC by performing statistical discrimination analyses; (iii) collect and geochemically analyse the soil samples; and (iv) develop and validate a heavy metal prediction model (HMPM) using soil metal concentration and spectral reflectance data. The study explores, for the first time, the potential of spectrally discriminating contaminant metals in floodplain soils, which has significant implications for the mapping and management of contaminated soils in mining-affected river catchments. The main research hypotheses were that: (i) soil spectra exhibit differences in specific wavelengths, which support their spectral discrimination; (ii) heavy metal concentrations can be retrieved from the spectra at high accuracy; and (iii) the samples with the highest heavy metal concentrations (high concentration of heavy metals means the colour of soil will be darker) would have the lowest reflectance (or the highest absorbance) and that reflectance would increase proportionally as heavy metal concentrations decreased.

## 2. Widespread Dispersal and Hazards of Heavy Metals in the UK 

Even though metal mining activity ceased almost a century ago, many west-draining rivers influenced by the flooding of June 2012 registered high concentrations of heavy metals in flood sediments that exceeded national and European standards [2]. Macklin et al. [4], Dennis et al. [42] and Brewer et al. [43] pointed out that at the catchments where historical metal mining took place, massive floods can cause dispersion, overbank sedimentation of highly contaminated constituents. In particular, deposition of fine-grained metal contaminated sediment on floodplains can pose a serious potential risk to the vigour, organisation and resilience of ecosystem services. Previous studies in the Ystwyth valley brought to light that sheep has the capacity to ingest high concentrations of heavy metals per day (1685 mg of Pb, 486 mg of Zn and 60 mg Cu), especially from the green vegetation during the winter [44,45,46].

The extraction of Pb, Zn and Cu from West Wales has a long history, linked back to the Roman period or the Bronze Age in some regions. Generally, Pb and Zn mining peaked in the mid-19th century, with most mining operations closed by the beginning of the 20th century [22,47,48,49,50].

Many European researches have reported that offal can hold an elevated concentration of metals. Rodríguez-Estival et al. [51] unexpectedly discovered that 91.4% of cattle and 13.5% of sheep had high blood Pb concentrations related to a subclinical vulnerability, and two cattle had blood Pb concentrations expressive of clinical poisoning. The previous studies are related directly with results from West Wales and presented the detailed risks of floodplain contamination from bovine species, which are found to be very vulnerable to Pb poisoning, especially young animals [52,53]. Furthermore, when the produced meat of the poisoned animals reaches the food chain, human health will be in danger. The problem of soil contamination by heavy metals in West Wales, and of course many other areas of the UK, will increase as a result of floods that happened in the past century. Therefore, innovative monitoring techniques, such as hyperspectral remote sensing, are highly recommended to characterise qualitatively and quantitatively the heavy metal contamination and investigate the short-term solutions, to protect the ecosystem services at large and human health specifically [2,28].

## 3. Materials and Methods

### 3.1. Study Area and Soil Sampling

**Figure 1:**
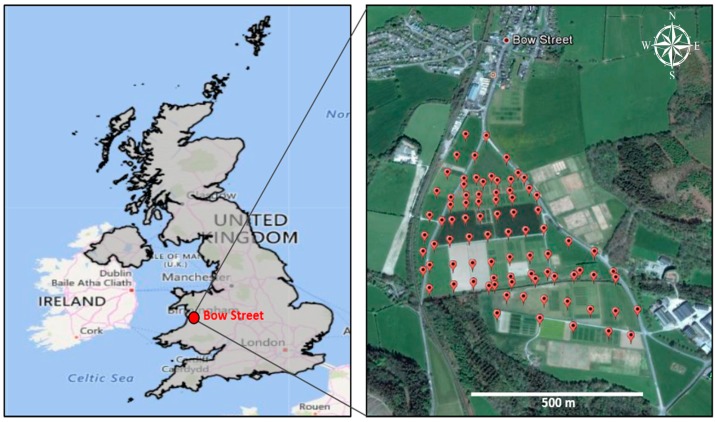
Geographical position of the study area and locations of the 85 sampling points.

The study area was Bow Street in West Wales, the UK. The site comprises a triangular plot of about 40 ha of land, divided into a series of experimental plots managed by Aberystwyth University’s Institute of Biological, Environmental and Rural Sciences (IBERS). The main land cover of the study area is forage crops that are used for grazing of sheep or cattle (Figure 1).

A total of 85 surface soil samples (0–5 cm) were collected from the study site using a stainless-steel trowel (Figure 1). After removing the vegetation from the soil surface, each soil sample of about 500 g was an aggregation of five individual spot samples obtained from a 1 m^2^ area. Samples were placed in wet-strength soil bags and, in the laboratory, they were oven-dried for 48 h at 40 °C. The reference for the soil samples locations was the work performed by Foulds et al. [2], who studied the contaminated area and found that flood sediments were polluted at a higher level of contamination guidelines. More importantly, crop silage harvested from the flood affected region was found to cumulate up to 1900 mg·kg^−1^ of sediment-associated Pb, which caused cattle poisoning and mortality. Two years later, in 2014, authors joined the research group of Paul Brewer and Mark Macklin at Aberystwyth University and have planned together to build on their work by testing the hyperspectral imaging method that has not been used before on this polluted site.

### 3.2. Field and Laboratory Spectral Measurements

Field spectra were acquired during August 2014 using ASD (Analytical Spectral Devices) FieldSpec^®^ 3 portable spectroradiometer in a hand-held mode. The instrument has a spectral resolution of 3 nm in the 350–1000 nm range and 10 nm in the 1001–2500 nm range, both ranges interpolated to 1 nm during the measurements. The field spectral measurements took place before taking the soil samples from the 85 locations. After removing the surface vegetation, five spectral measurements were performed directly from each sample location and averaged to a single representative spectrum. Later on, the soil samples were collected following the exact spots (each location has 5 spots) used during the spectral measurements. In the field, the sun was the only source of illumination and the measurements were taken from 10 AM to 2 PM under clear sky conditions. White panel reference data were recorded before each soil measurement. Before starting the measurements, a warm up time of 30 min was given to minimise errors caused by the warming of the spectroradiometer array. To collect the spectra, a pistol grip was pointed towards the soil at 50 cm height. The radius of the field-of-view (FOV) was 3.5 cm, as estimated using Equation (1), where R is the radius of the FOV, H is the height from the soil to the sensor in the pistol grip and AOV is the angle-of-view of the sensor (8°).

R = tg(AOV/2) × H × 100 [cm](1)

**Figure 2:**
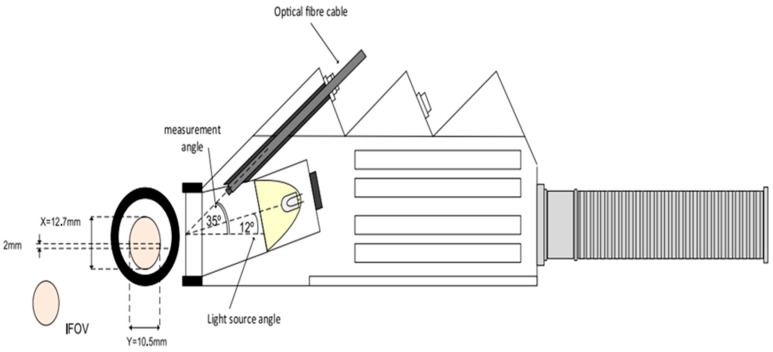
Illustration of the ASD high-intensity contact probe according to ASD Inc [54]. X and Y are the height and width, respectively, of the Field of View (FoV).

**Figure 3:**
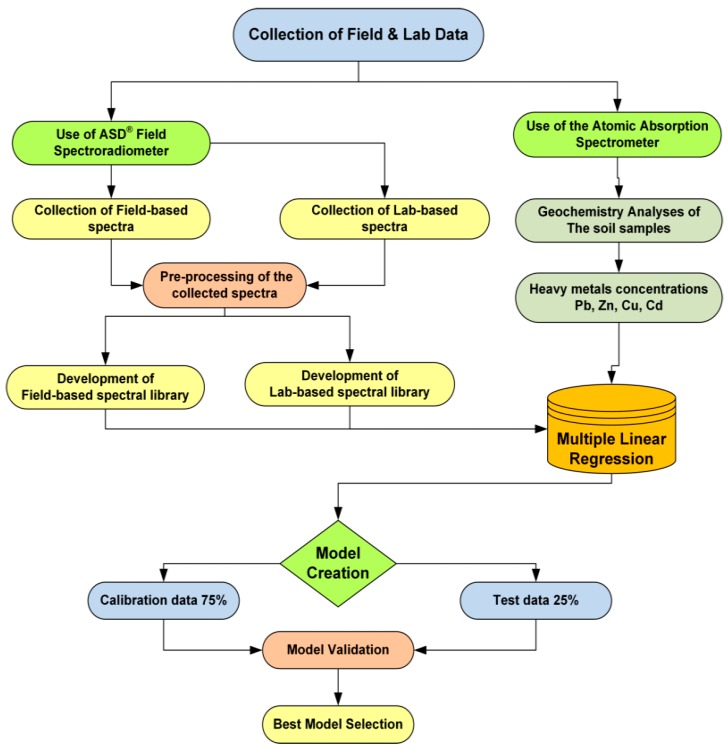
Flowchart showing the methodology steps implemented in this study.

In the laboratory, the collection of soil spectra was performed with a high-intensity contact probe (CP; direct contact with the soil). The ASD CP setup has a 100 W reflectorised halogen lamp aligned at 12° to the probe body, and the sensed spot has a diameter d_probe_ = 1.1 cm with a FOV = 1.33 cm^2^. Analogous to the field spectra, before starting the measurement, a warm up time of 30 min was respected. To collect the soil spectrum, soil (particle size < 2 mm) was placed in a black plastic dish (size of Petri dish) and the ASD CP was put in a direct contact with the soil, followed by registration of the spectrum. Each measurement was repeated three times and averaged to representative spectrum per sample (Figure 2 and Figure 3).

### 3.3. Geochemistry Analysis of the Soil Samples

The extraction of heavy metals (Cd, Cu, Pb and Zn) was performed using nitric acid. A number of acids and acid mixtures (e.g., hydrofluoric-perchloric-nitric or perchloric-nitric) are efficient in decomposing samples of rock, soils or sediments. Concentrated nitric acid alone has a less vigorous effect than mixed acid decomposition methods and, in particular, iron (Fe (III)) oxide minerals are not attacked strongly. However, the following procedure is an efficient method for metals such as Cd, Cu, Pb and Zn. Regarding the geochemistry analysis, the soil samples were put in the oven to dry at 40 °C. Soil samples with small particle size (< 63 μm) were examined in the laboratory, as this fraction category usually shows the highest concentration with metals [55]. 

For the 85 soil samples, the geochemistry analysis followed these steps: (1) weigh 0.5 ± 0.005 g of soil using a weighing boat and transfer each sample into a clearly labelled boiling tube; (2) use of an automatic dispenser to add 2 ml of concentrated nitric acid carefully to the sample; (3) place the test tube carefully into the digestion block that has been exposed to 100 °C and leave it for 1 h, (4) take the boiling tube out of the block and leave to cool; (5) use an automatic dispenser to add 18 ml of distilled water to the contents of the tube and mix thoroughly with a whirlimixer (Fisher Scientific Ltd., Loughborough, UK); and (6) leave overnight (covered in cling film) to allow any suspended particles to settle. After that, the samples can be carefully sprayed into the flame of an atomic absorption spectrometer (PerkinElmer Inc., Shelton, CT, USA) without blocking the capillary tube. The dilution factor for this method is 40, but in cases where the concentration was higher than the calibration range of the spectrometer, the samples were diluted using an automatic dilutor by preparing serial dilutions of X 10. For the aim to control the analytical methods applied in the current study, certified reference material (GBW 07307 stream sediment) was made ready and analysed following identical steps as used for HMSC.

### 3.4. Data Processing and Statistics

The obtained field and lab spectra were continuum-removed and normalized to increase the spectral absorption features. The continuum-removal analysis suggested by Clark and Roush [56] is the standard transformation in land cover spectral discrimination [57,58,59]. The continuum is a convex hull of straight-line segments, fitted over a spectrum and subsequently removed by division or rationing relative to the spectrum [60].

Since not all wavelengths are adequate for detection of heavy metals in soils, analysis of variance (ANOVA) was conducted first to find out broader spectral bands sensitive to heavy metal concentrations. This analysis was at each wavelength from 350 to 2500 nm for each spectral library (field- and lab-based) at 95% confidence level. ANOVA was followed by correlation analysis between the spectral features and heavy metal concentrations following the Pearson’s correlation coefficient. The latter give an excellent estimation of dependence between two quantities, and it is calculated after dividing the covariance of the two variables by the product of their standard deviation. The correlation coefficient can be estimated by Equation (2), where E is the expected value, *μ* is the mean, *cov* is the covariance between x and y and *corr* is the correlation coefficient [37].

(2) $$Corr\left(X,Y\right)=\frac{Cov\left(X,Y\right)}{\sigma X\sigma Y}=\frac{E[\left(X-\mu x\right)\left(Y-\mu y\right)}{\sigma X\sigma Y}$$

High correlation coefficient between the independent variable (spectral features) and the dependent variable (heavy metal concentrations) indicates a strong linear correlation. The correlation was conducted at 95% confidence level. This was followed by stepwise multiple linear regression (SMLR) to create the HMSC models. For the SMLR, at each step the independent variable (wavelengths) not in the equation and with the smallest probability of F is entered, provided that the probability is sufficiently small. Variables in the regression equation are removed if their probability of F becomes sufficiently large. The method stops when no more variables are eligible for inclusion or removal [61]. The generated regression models were written following Equation (3):

HMSC (mg kg^−1^) = [A_n_*R*_350–2500_ + B] × 1000 (3)

where HMSC is the heavy metal soil concentration (mg kg^−1^), A_n_ is the slope of the regression (n coefficients of the regression), *R*_350–2500_ is the reflectance wavelength varying from 350 to 2500 nm, B is the regression constant and we multiply the result by 1000 in order to obtain the concentration of heavy metal with mg per kg (mg kg^−1^). The overall methodology framework is depicted on Figure 3.

## 4. Results and Discussion

### 4.1. Soil Descriptive Statistics

**Figure 4:**
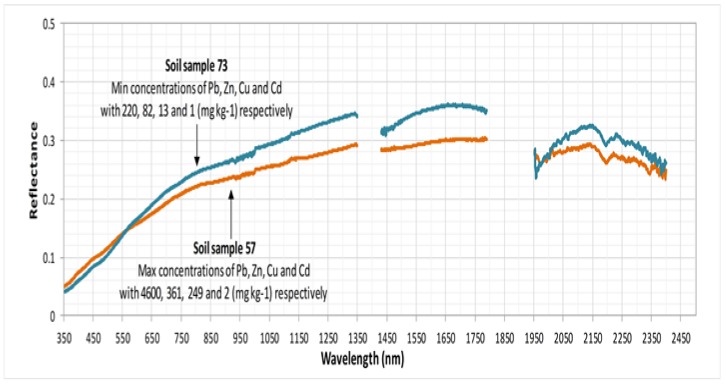
Mean spectra for soil samples characterised by low (sample 57) and high (sample 73) concentrations of heavy metals in the study site.

Pb, Zn, Cu and Cd are the major heavy metals in the study area [2], thus they were selected as potential contaminants. The descriptive statistics of the geochemistry analyses are summed up in Table 1. The standard deviation (Stdev) of Pb and Zn was very high (1037.96 and 59.85, respectively), indicating large spatial variability of these two metals and existence of “hot spots” (i.e., localized small areas with their very high concentration; Figure 3 and Figure 4). The concentrations of Cu and especially Cd showed lower magnitude and variation. As Pb was found in the highest concentration compared to the other heavy metals, its concentration might be significantly responsible for the variation of the soil reflectance. 

**Figure 5:**
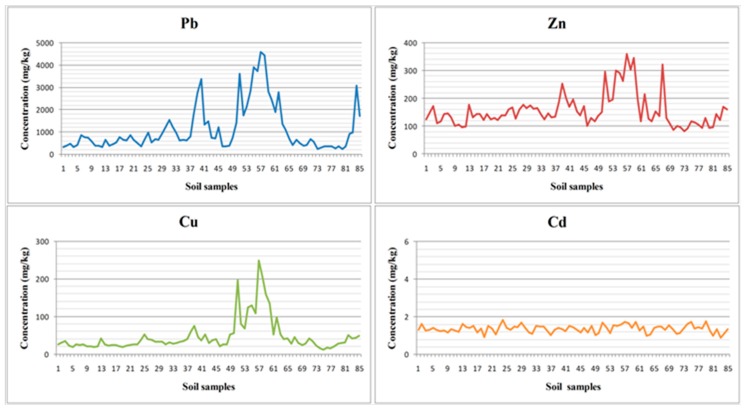
Mean (n = 85) variation in concentrations of the four heavy metals found in the study site.

Figure 4 below tends to support the third hypothesis by showing lower reflectance of the highly contaminated soil sample (sample 57) compared to the reflectance of the low contaminated soil sample (sample 73), although more studies are needed to further support this. Herein, it can be clearly noted that the reflectance was governed by the concentration of heavy metals in each sample. For sample 57, which had the maximum concentration of the four heavy metals, the reflectance was lower than sample 73, which had the minimum concentration (Figure 5).

### 4.2. Development of Field- and Lab-Based Spectral Libraries

**Figure 6:**
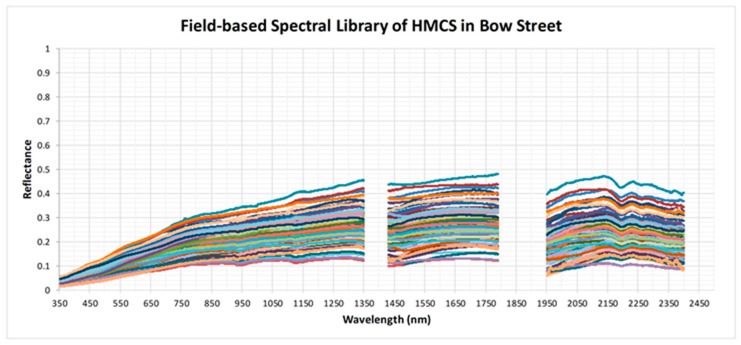
Field-based spectral library of heavy metal soil contamination (HMSC) at the Bow Street site. Spectral regions related to water vapor absorption (1350–1430, 1790–1950 and 2400–2500 nm) have been removed.

**Figure 7:**
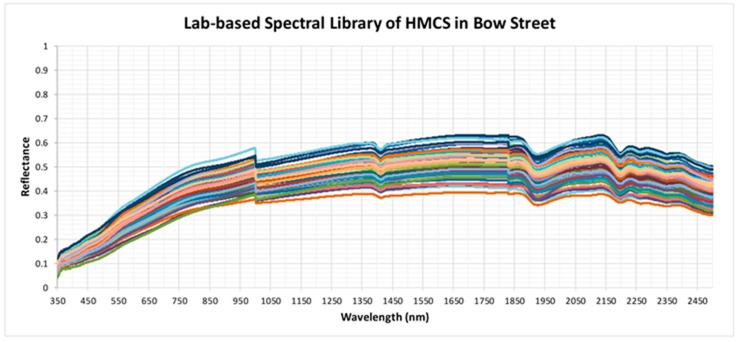
Lab-based spectral library of the heavy metal soil contamination (HMSC) at the Bow Street site.

The field- and the lab-based soil spectral libraries, composed of 85 spectra each, are shown on Figure 6 and Figure 7, respectively. Overall, the soil spectra resembled the typical shape of an increase in the VIS and relatively constant in the NIR and SWIR, with some local dips around 1400, 1900 and 2200 nm due to water and clay absorption. Yet, major variation in the soil reflectance for both libraries could be seen in the NIR and especially SWIR, likely due to variation in soil properties such as moisture, clay and organic matter content, but also due to content of heavy metals. The two spectral libraries enrich the spectra database and may serve as “reference” spectra for heavy metal contaminated soils in the UK, and; thus, authorise appropriate validation of the reflectance information extracted from radiance data acquired from remote platforms, and can play an important role in tracking temporal changes of the soil spectra over the sampling locations. 

### 4.3. Statistical Discrimination Analysis 

**Figure 8:**
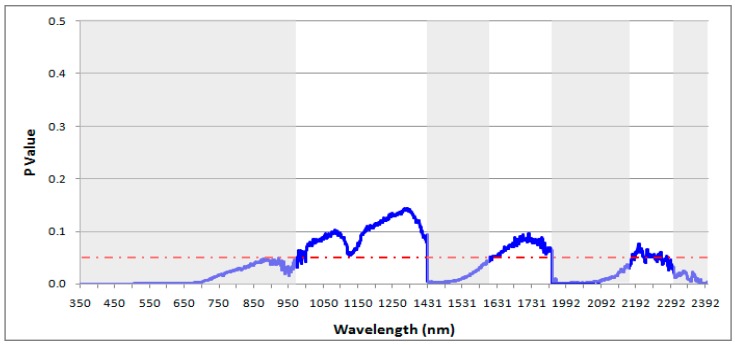
Wavelength-intervals shaded grey depict statistically significant differences between the field-based spectra. The red-dashed line denotes the limit for statistical significance (95% confidence level).

**Figure 9:**
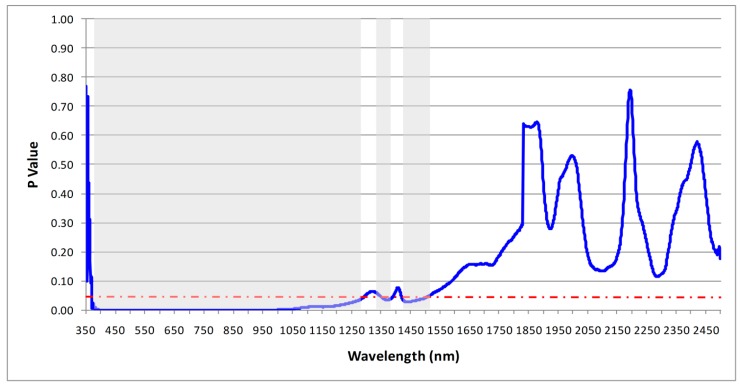
Wavelength-intervals shaded grey depict statistically significant differences between the lab-based spectra. The red-dashed line denotes the limit for statistical significance (95% confidence level).

The results of the ANOVA on the continuum-removed reflectance at each wavelength, from 350–2500 nm, are plotted in Figure 8 and Figure 9, for the field- and the lab-based spectral libraries, respectively. The results are based on the continuum removed spectra of the soils and the red-dashed line on the figures denote the critical P value (0.05), below which statistically significant results are achieved (shaded grey on the figures, i.e., spectral regions that contain at least one significantly different soil spectral from the others).

The results showed that there were statistically significant differences in the mean continuum-removed field-based soil spectra along most of the VIS spectrum, from 350 to 800 nm, and narrower windows in the NIR and SWIR. For the lab-based soil spectra, the majority of the statistically significant results could be seen in the VNIR, from 360 to 1270 nm, with few narrow windows at the end of the NIR. Yet, ANOVA cannot reduce the number of wavelengths to those most influential for discriminating between the soils, but can serve as an input for further statistical modelling of HMSC.

### 4.4. Model Development and Validation

The coefficient of determination (R^2^, the square of the Pearson correlation coefficient) for the SMLR models shows information about useful spectral bands that were mostly used in building of the models; the selected wavebands used to build the field-based and lab-based prediction models are listed in Table 2 and Table 3, respectively. In the regression analyses, the spectral bands were considered as an independent variable, whereas the concentration of heave metals were implicated as a dependent variable. For every single step in the SMLR, the non-included independent variable with the lowest probability is integrated, only if its probability is small. Finally, the performance quality for each calibration model was evaluated by R^2^. Song et al. [37] built models to assess aluminium, copper and chrome in the soil and water of a mining area in China. These authors derived, from measured spectra, spectral features characteristic for their metals. More importantly, they also found notable linear correlation between spectral wavebands and heavy metal concentrations. Therefore, the bands spectra selected from field- and lab-reflectance spectra are involved in the construction of the prediction models. Liu et al. [62] investigated Cd and Pb concentrations spectroradiometrically in Chinese soils and, based on SMLR, achieved R^2^ for estimating the heavy metal contents of about 0.65–0.82 for Cd and 0.78 to 0.88 for Pb. The R^2^ values reported in our study were somewhat similar and sometimes better than previous studies; the predictive power of the models may be tested and possibly improved by using unaltered or transformed spectra (e.g., logarithm of reciprocal spectra; [62]; derivative spectra; [39]) and other statistical models (e.g., generalized regression neural network; [63]).

Based on Table 2, the four developed field-based HMPMs were:

Pb_FSpec_ = [−320.758*R*_354_ + 456.742*R*_389_ − 94.144*R*_582_ + 92.316*R*_1719_ − 82.081*R*_1775_ − 0.172] × 1000

Zn_FSpec_ = [−64.043*R*_366_ + 71.865*R*_374_ − 57.897*R*_386_ + 90.868*R*_388_ + 66.374*R*_393_ − 96.782*R*_394_ − 6.142*R*_586_ + 0.965*R*_1348_ + 0.139] × 1000

Cu_FSpec_ = [−81.125*R*_367_ + 42.275*R*_368_ + 64.551*R*_389_ − 23.652*R*_434_ + 0.026] × 1000

Cd_FSpec_ = [0.008*R*_1951_ − 0.007*R*_1978_ + 0.001] × 1000

Based on Table 3, the four developed lab-based HMPMs were:

Pb_LSpec_ = (90.729*R*_356_ − 25.105 *R*_618_ − 0.057) × 1000

Zn_LSpec_ = (−4.369 *R*_358_ + 5.055 *R_368_* + 9.101 *R*_376_ − 78.747 *R*_470_ + 127.870 *R*_475_ − 53.910 *R*_484_ − 0.048) × 1000

Cu_LSpec_ = (2.502*R*_359_ − 0.628*R*_651_ − 0.016) × 1000

Cd_LSpec_ = (−0.001 *R*_1465_ + 0.002) × 1000

Many of the wavelengths correlated with the soil heavy metal concentrations were in the VIS bands, probably due to molecules in the soil responsible for some traits characterising this region, such as organic matter content and its various chemical functional groups. Reflectance (i.e., absorbance) bands are generally caused by fundamental molecular vibrations; yet, most heavy metals do not present specific spectral features in the NIR and SWIR, so understanding of the physical relationship between the spectral data and the heavy metals is not straightforward and is probably influenced by presence/absence of other inorganic components, such as iron cations and phosphate and carbonate anions [64]. It is; therefore, primarily the mathematical relations (e.g., HMPM) that can be used for prediction, testing and calibration/validation purposes [64,65].

The ASD field spectroradiometric data are non-destructive and efficient for estimation of heavy metal levels in the soil. High correlation between the estimated heavy metal concentrations and the predicted heavy metal contents highlights the feasibility of using SMLR to build reliable predictive models with spectral measurements and geochemical variables from laboratory analyses [62,66,67]. Furthermore, there is no need for chemicals reagents, and it requires minimal sample preparation. The present research contributes to the growing field of hyperspectral imaging by advancing the knowledge on how a combination of field spectroradiometric data with geochemical analyses can be used to predict heavy metal contamination and to assess better the environmental quality.

## 5. Conclusions

The present research clearly highlighted the potential role of combining field and laboratory hyperspectral data with geochemical data of Pb, Zn, Cu and Cd in order to quantify and model heavy metal contamination by considering a highly contaminated floodplain site from Wales in the west of the UK. The results confirm the pre-defined study hypotheses: first, that soil spectral signatures exhibit differences in specific wavelengths of the spectrum, thus supporting their spectral discrimination; second, that heavy metal concentrations can be retrieved from spectral reflectance data at reasonable accuracy, using field spectroradiometer covering the spectral range of 350–2500 nm; and third, that the samples with the highest heavy metal concentrations would have the lowest reflectance and that reflectance would increase as heavy metal concentrations decreased. 

Herein, the field- and lab-based spectral features derived from 85 soil samples of the contaminated area were used successfully to develop two spectral libraries, which have been combined to build eight heavy metal prediction models using SMLR. The findings demonstrate high feasibility to predict HMSC in a highly contaminated floodplain site by combining soil geochemistry analyses and spectroradiometry. 

The problem of heavy metal contamination in West Wales and several other areas in the UK can be characterised effectively using hyperspectral spectroradiometry, which has the potential as a rapid, low cost technique for mapping HMSC. However, as the spectral features of soil in the spectral range from 350–2500 nm is very complex, finding exact spectral wavebands attributable to Pb, Zn, Cu and Cd concentrations, which are unaffected by the chemical composition and physical conditions at the soil surface, is a significant challenge. The developed prediction models provide an alternative tool for predicting the heavy metal contamination by using field and laboratory hyperspectral measurements. The produced models can be a basis for mapping heavy metal concentrations over a large area by using space-borne hyperspectral sensors such as Hyperion, AVIRIS, EnMAP and CHRIS Proba.

## Figures and Tables

**Table 1:** Descriptive statistics of heavy metal concentrations in the soil of the contaminated area in Bow Street, UK, based on 85 samples. Max, Min, Median, Mean and Stdev are maximum, minimum, median, mean and standard deviation, respectively. The minimum detection limits of the atomic absorption spectrometer were 0.8, 1.5, 1.5 and 15 mg kg^−1^ for Cd, Cu, Zn and Pb, respectively.

mg kg^−1^	Pb	Zn	Cu	Cd
Max	4600	361	249	2
Min	220	82	13	1
Median	670	140	32	1
Mean	1100	156	47	1
Stdev	1037.959	59.850	42.869	0.204

**Table 2:** Summary of the selected spectral bands and regression coefficients for the field-based spectral library using stepwise multiple linear regression. A dash denotes that the spectral band was not included in the model equation for the relevant heavy metal.

Spectral Bands	Model Coefficients for the Studied Heavy Metals			
	Pb	Zn	Cu	Cd
354 nm	−320.758	-	-	-
366 nm	-	−64.043	-	-
367 nm	-		−81.125	-
368 nm	-	-	42.275	-
374 nm	-	71.865	-	-
386 nm	-	−57.897	-	-
388 nm	-	90.868	-	-
389 nm	456.742	-	64.551	-
393 nm	-	66.374	-	-
394 nm	-	−96.782	-	-
434 nm	-	-	−23.652	-
582 nm	−94.144	-	-	-
586 nm	-	−6.142	-	-
1348 nm	-	0.965	-	-
1719 nm	92.316	-	-	-
1775 nm	−82.081	-	-	-
1951 nm	-	-	-	0.008
1978 nm	-	-	-	−0.007
Constant	−0.172	0.139	0.026	0.001
R^2^	0.671	0.697	0.561	0.123

**Table 3:** Summary of the selected spectral bands and regression coefficients for the lab-based spectral library using stepwise multiple linear regression. A dash denotes that the spectral band was not included in the model equation for the relevant heavy metal.

Spectral Bands	Coefficients of the Four Heavy Metals			
	Pb	Zn	Cu	Cd
356 nm	90.729	-	-	-
358 nm	-	−4.369	-	-
359 nm	-	-	2.502	-
368 nm	-	5.055	-	-
376 nm	-	9.101	-	-
470 nm	-	−78.747	-	-
475 nm	-	127.870	-	-
484 nm	-	−53.910	-	-
618 nm	−25.105	-	-	-
651 nm	-	-	−0.628	-
1465 nm	-	-	-	−0.001
Constant	−0.057	−0.048	−0.016	0.002
R^2^	0.641	0.642	0.428	0.048
